# Influence of Fermentation on Functional Properties and Bioactivities of Different Cowpea Leaf Smoothies during In Vitro Digestion

**DOI:** 10.3390/foods12081701

**Published:** 2023-04-19

**Authors:** Mapula R. Moloto, Stephen A. Akinola, Faith Seke, Tinotenda Shoko, Yasmina Sultanbawa, Jerry L. Shai, Fabienne Remize, Dharini Sivakumar

**Affiliations:** 1 Phytochemical Food Network Group, Department of Crop Sciences, Pretoria 0001, South Africa; mapularebahlotsem@gmail.com (M.R.M.); akinolasa@tut.ac.za (S.A.A.); fayeesk@gmail.com (F.S.); shokot@tut.ac.za (T.S.); 2 Australian Research Council Industrial Transformation Training Centre for Uniquely Australian Foods, Queensland Alliance for Agriculture and Food Innovation, Centre for Food Science and Nutrition, The University of Queensland, Elkhorn Building (#1024), 80 Meiers Road, Indooroopilly, Brisbane, QLD 4068, Australia; y.sultanbawa@uq.edu.au; 3 Department of Biomedical Sciences, Tshwane University of Technology, Arcadia, Pretoria 0001, South Africa; shailj@tut.ac.za; 4 SPO, Université de Montpellier, Université de La Réunion, Institut Agro, INRAE, 2 Place Viala, F-34000 Montpellier, France

**Keywords:** in vitro gastrointestinal digestion, antioxidant properties, carotenoids, postharvest processing

## Abstract

This study investigated the effects of *Lactiplantibacillus plantarum* 75 (LAB 75) fermentation at 37 °C for 48 h on the pH, total soluble solids (TSS), colour, total titratable acidity (TTA), carotenoids, and bioactivities of cowpea leaf smoothies from three cultivars (VOP 1, VOP 3, and VOP 4). Fermentation reduced the pH from 6.57 to 5.05 after 48 h. The TTA increased with the fermentation period, whilst the TSS reduced. Fermentation of the smoothies resulted in the least colour changes (∆E) in VOP 1 after 48 h. Fermentation of cowpea smoothies (VOP 1, VOP 3, and VOP 4) improved the antioxidant capacity (FRAP, DPPH, and ABTS), which was attributed to the increase in total phenolic compounds and carotenoid constituents in all of the fermented cowpea smoothies. VOP 1 was further selected for analysis due to its high phenolic content and antioxidant activity. The VOP 1 smoothie fermented for 24 h showed the lowest reduction in TPC (11%) and had the highest antioxidant (FRAP, DPPH, and ABTS) activity. *Ltp. plantarum* 75 was viable and survived the harsh conditions of the gastrointestinal tract, and, hence, could be used as a probiotic. VOP 1 intestinal digesta showed significantly higher glucose uptake relative to the undigested and the gastric digesta, while the gastric phase had higher levels of α-amylase and α-glucosidase compared to the undigested samples.

## 1. Introduction

Cowpea (*Vigna unguiculata*) leaf consumption is very common in Africa and the leaves have been reported to be part of the food security crops. Cowpea leaves are a very good source of polyphenols and carotenoids [1,2]. In a previous study, digestion affected the stability of cowpea carotenoids, namely, α-carotene, all-trans β-carotene, zeaxanthin, 9-cis-β-carotene, and lutein. When compared to undigested cowpea leaves, antioxidant activity was lowered in the intestinal fraction during digestion (VOP 1, VOP 4, and VOP 3). The strong antidiabetic properties of cowpea leaves are associated with their high levels of expression in the glucose transporter genes in muscle cells [1,2]. Polyphenols from cowpea leaf extract were found to have strong inhibitory properties against α-amylase and α-glucosidase [2]. The aforementioned enzymes are crucial in regulating obesity and blood glucose levels because of their ability to restrict the re-absorption of glucose in the gut. The inhibition of carbohydrate hydrolysing enzymes (α-amylase and α-glucosidase) was significantly lessened during digestion [2]. Zeaxanthin, all-trans-β-carotene, α-carotene, 9-cis-β-carotene, and lutein in cowpea cultivar leaves were linked to antioxidant properties and an inhibitory effect on α-amylase and α-glucosidase activity [2]. 

Indigenous African leafy vegetables contribute significantly to Africa’s food security, especially in rural and peri-urban settings. However, a significant number of indigenous vegetables are lost due to improper and inadequate storage, packaging, transport, and handling technologies [3,4]. Various postharvest preservation techniques have been developed and these include blanching, air-drying, and solar drying [4,5]. Food drying could typically lead to product degradation from all vantage points, including sensory, physicochemical, and nutritional. Conventional drying techniques are more prone to mechanical and chemical deterioration in the finished product [6]. As a result, it is critical to consider other postharvest techniques for fruits and vegetables and to select appropriate conditions that will minimize potential changes. One of the techniques that have been developed but have received little to no attention is green vegetable fermentation. Fermentation is thought to be a straightforward and efficient biotechnology process that aids the preservation of vegetable safety and sensitivity and improves nutrition and shelf life. Due to its benefits for cost-effectiveness and environmental protection, fermentation is used extensively in food preservation around the world [7]. The health-promoting qualities of fermented vegetable products include antioxidant activities, improved gastrointestinal health, antibacterial activities, reduced cardiovascular risk factors, and anti-inflammatory action. These advantages of fermented vegetables could be attributed to the present carotenoids and phenolic compounds [8]. Traditional green vegetables are fermented in Africa, but are less common than cereals, tubers, meat, milk products, and alcoholic beverages [9]. 

Lactic acid fermentation is one of the traditional and affordable methods of food preservation and protection in rural settings [10]. During the fermentation process, food carbohydrates are converted into acid and other by-products by lactic acid bacteria (LAB). Lactic acid fermentation improves the nutritional components and the sensory qualities, such as aroma, flavour, and consumer acceptability [11]. Bartkiene et al. [12] reported the effects of lactic fermentation on tomato pulp using *Lactobacillus sakei KTU05-6*, *Pediococcus acidilactici KTU05-7*, and *Pediococcus pentosaceus KTU05-8* as inoculum. The lacto-fermentation process affected the amount and the cis/trans ratio of lycopene and α-carotene, resulting in a higher cis/trans lycopene ratio. The higher cis-lycopene isomers are preferentially micellarized due to being less prone to crystallization, oil solubility, and quick absorption by the intestinal cells compared to the all-trans forms [13,14]. In comparison to raw nightshade leaves, the fermentation of nightshade leaves with *Ltp. plantarum* 75 showed that fermentation increased the concentration of ascorbic acid after three days, reduced colour changes, and increased the total polyphenol content and antioxidant activity [15]. Managa et al. [16] also found that *Ltp. plantarum* 75 boosted the phenolic content and antioxidant activity of chayote leaf and pineapple smoothies after fermentation. Gao et al. [17] reported improved total phenol, polyphenols, and antioxidant capacity in an indigenous vegetable *Momordica charantia* after lactic acid fermentation. On the other hand, the ratio of a molecule’s availability following gastrointestinal digestion to its availability before digestion is used to assess a molecule’s potential usefulness. Due to oxidation or polymerization processes, the pH changes that take place during the gastrointestinal digestion phases result in phenolic derivatives with a high molecular weight and poor solubility that are inaccessible for absorption [18]. With the help of lactic acid bacteria, polyphenols can thus be biotransformed into molecules with improved bioavailability and bioactivity [19]. Zhao et al. [20] found that in vitro digestion and fermentation by lactic acid bacteria in tea extracts increased the antioxidant activity and cellular absorption of phenolic components. This was also reported with pomegranate juice [21] and kiwi fruit pulp [22] as well as Korean leek and cowpea leaves [23,24]. 

This work contributes by enhancing the low-cost fermentation method for the improved utilization of cowpea leaves, which are already available in rural communities, thereby making a case for its commercialization. Furthermore, when fewer optimum processes are used in cowpea leaf fermentation, the improved sensory qualities and health benefits will drive consumer approval. As a result, there appears to be an opportunity to increase efforts in Africa to study and adopt this type of biological preservation strategy for leafy vegetables. In light of the abovementioned, the objectives of this study were to ascertain the impact of the fermentation of *Ltp. plantarum 75* on the physicochemical properties of fermented cowpea leaf smoothies and to investigate the changes in the total phenol and carotenoid components, inhibition of carbohydrate hydrolysing enzymes (α-amylase and α-glucosidase), antioxidant properties (FRAP, DPPH, and ABTS), and the bioaccessibility of the fermented smoothies following an in vitro gastrointestinal digestion.

## 2. Materials and Methods

### 2.1. Chemicals and Reagents

Biokar Diagnostics (Solabia company, Pantin, France) and Conda Laboratories (Madrid, Spain) supplied the culture media. The LAB strain used in this study (*Ltp. plantarum 75* )was obtained from the culture collections of the microbiology laboratory at the QualiSud, Université de La Réunion, France. All chemicals and reagents used in this study were of analytical grade and were obtained from Sigma Aldrich (Johannesburg, South Africa), and HPLC-grade reagents (≥99.8%) were used for the UHPLC analysis.

### 2.2. Plant Samples

Cowpea cultivars (VOP I, VOP 3, and VOP 4) were planted and propagated at Tshwane University of Technology in Pretoria, South Africa, as detailed by Moloto [1]. Each cowpea cultivar was planted in a randomized five-replicate configuration. Irrigation was carried out at a rate of 100 mL per day. At the 8-leaf growth stage, clean leaves were plucked and cleaned with tap water.

### 2.3. Preparation and Fermentation of Smoothie

The VOP 1, VOP 3, and VOP 4 cowpea fresh leaves were rinsed with tap water containing 0.01% calcium hypochlorite to remove dirt and soil before cleaning in sterile distilled water. Leaves were allowed to drain and were dried on a paper towel before blending into smoothies in a Russell Hobbs blender. The mixture was then pasteurized for 10 min in a water bath at a core temperature of 60 °C and cooled to room temperature for 2 h before fermentation [15]. 

### 2.4. Reactivation of the Ltp. plantarum 75 Cultures and Fermentation of Cowpea Smoothies

The *Ltp. plantarum 75* was reactivated, and the smoothies were fermented according to Mashitoa [18]. The LAB culture was reactivated in MRS broth overnight at 30 °C, inoculated into fresh MRS broth, and incubated for 48 h at 30 °C. The broth culture was centrifuged at 8000× *g* for 5 min, and the cells were washed in sterile saline water. At 660 nm, the cell population was determined in a UV-Spectrophotometer, and the cell population was adjusted to 0.05 McFarland standard concentrations (6 Log CFU/mL). One (1) mL of the *Ltp. plantarum* 75 culture (6 Log CFU/mL) was inoculated into 100 mL of smoothies and incubated at 37 °C for 2 days. During the incubation period, the smoothies were withdrawn for analysis at 2, 24, and 48 h. The fermentation of smoothies using different cultivars was performed in triplicate.

### 2.5. Physicochemical Properties of Fermented and Unfermented Cowpea Smoothies

Only pasteurized and fermented smoothies had their physicochemical parameters tested at 0, 2, 24, and 48 h of fermentation. The pH of the samples was determined using the EUTECH pH2700 Instrument (EUTECH Instruments, Illinois, IL, USA), and the total soluble solids (TSS) was determined using the ATAGO PAL-3 pocket refractometer (Atago USA Inc., Tokyo, Japan). The refractive index values obtained were saved in Brix. The total titratable acidity of the samples was evaluated using the Reddy et al. [5] technique. The effect of fermentation on the colour characteristics of the smoothies was measured using a CM-3500 d spectrophotometer and spectral magic NX software (Konica Minolta, Konica Minolta Sensing Inc., Tokyo, Japan). The samples’ degree of brightness (L*), red-to-green component (a*), and yellow-to-blue component (b*) were all measured. The total colour difference (∆E) was calculated following Managa [15].

### 2.6. Organoleptic Properties of Unfermented and Fermented Cowpea Smoothie

A quantitative descriptive analysis technique, as described by Mashitoa [19], was used for the sensory evaluation of the smoothies, with some modifications. Ten trained panellists were selected from the pool of assessors trained to identify the desired characteristics of the smoothies. The panellists were composed of healthy male and female research employees. There were two training sections adopted, and the samples were rated using a structured scale ranging from 0 to 9 (Absent = 0, 1–3 = weak, 4–6 = moderate, 7–9 = strong). Coded samples were served chilled in white cups with lids to the panellists under a white light-illuminated cubicle. Panellists evaluated samples based on the agreed attributes of the smoothies, and the ratings of samples were converted into intensity scores. The colour perception was assessed using the light and dark colour perception in green leafy vegetables as a reference. The characteristics of aroma and flavour were assessed using veggie aroma in a common fresh leafy vegetable, The perception of texture in the smoothie was based on its viscosity in the mouth and was assessed using glucose syrup as reference. The assessment of sour taste and sweetness was based on the perception of tart and sweet taste using diluted citric acid and diluted sucrose solution, respectively, as references. A commercial fermented vegetable smoothie was used as a reference to determine the overall acceptability of smoothies. 

### 2.7. Total Sugars of Cowpea Smoothies

Total sugars were evaluated using a method described by Nielsen [25]. The polysaccharide was hydrolysed with concentrated sulfuric acid to yield hydroxyl methyl furfural, which was then condensed with phenol to yield a stable yellow-gold solution. To calculate the total carbohydrate content, 1 mL of each test or standard solution was added to a test tube. Then, 1 mL of a 5% phenol solution was introduced. A mechanical pipet was used to swiftly add 5 mL of concentrated sulfuric acid to the entire solution while it was being swirled in a vortex mixer. The mixture was then immediately combined and allowed to react for 10 min. At this point, the solution absorbance was read at 488 nm using a microplate reader (CLARIOstar Plus BMG Labtec, Lasec, Cape Town, South Africa). 

### 2.8. Carotenoid Extraction, Identification, and Quantification

The carotenoids were extracted using a method described by Moloto et al. [1]. From each smoothie powder, 5 g of samples were mixed with 4 mL of acetone, 95% ethanol, and 0.1% butylated hydroxytoluene (*w*/*v*). The samples were centrifuged for 10 min at 2500 rpm (Eppendorf 5804R Centrifuge, Hamburg, Germany), and the supernatant was collected. The extraction procedure was repeated four times with a 70:30 *v*/*v* mixture of hexane and dichloromethane containing 0.1% butylated hydroxytoluene. The extracts were freeze-dried and kept at a temperature of 80 °C. An HPLC–UV–DAD system (Shimadzu, Kyoto, Japan) was utilized to identify the individual carotenoids. The dried extracts were diluted in methanol, methyl tert-butyl ether (50:50, *v*/*v*) containing 0.1% butylated hydroxytoluene, before analysis. Chromatographic separation was performed on a YCM C30 carotenoid column (3.6 × 250 mm, 3.6 µm) (Waters, Milford, MA, USA) maintained at 25 °C, with a mobile phase consisting of 0.1% formic acid in methanol (solvent A) and 0.1% formic acid in MTBE (solvent B). The gradient program was used and the program was run as described: 0 min, 80%, 20 min, 75%, 30 min, 30%, 33 min, 30%, and 36 min, 80%, at the flow rate of 0.6 mL/min. Carotenoid standard concentrations (0 to 60 ppm) of lutein, zeaxanthin, α-carotene, 9-cis-β-carotene, and trans-β-carotene were used to quantify the carotenoid profiles in the smoothies. The calibration curves, the limit of detection (LOD), and the limit of quantification (LOQ) are provided in the Supplementary Data, Table S1.

### 2.9. In Vitro Digestion of Cowpea Smoothies

The in vitro digestion of the cowpea leaf smoothies was performed on the VOP 1 cultivar. The VOP 1 cultivar was selected based on its high total phenols, antioxidant activities, and carotenoid profile after fermentation for 24 h. Using a technique previously reported by Seke et al. [2], an amount of simulated salivary fluid (10 mL) at pH 7 containing 75 U mL^−1^ of α-amylase enzyme was added to 10 g of VOP 1, and the mixture was homogenized with a pestle and mortar for 10 s to mimic chewing before being incubated in a shaking water bath at 170 rpm for 2 min at 37 °C. The simulated gastrointestinal fluids were prepared, as described by Seke et al. [2]. Simulated gastric fluid (20 mL) was added to the oral digesta, and the gastric phase was initiated by adjusting the pH to 2.5 by adding 6 M HCl and pepsin solution (2000 U mL^−1^ in 0.1 M HCl, pH 2.2). The mixture was stirred at 170 rpm for 2 h at 37 °C, and 10 mL samples were collected and cooled on ice for 10 min to stop reactions and then stored at 80 °C. The intestinal and dialysis phases were initiated by adding the simulated intestinal fluid (20 mL) to the remaining gastric digesta, and the pH was adjusted to 7.5 using 2 M NaOH. The intestinal digesta was transferred into a dialysis tube (10 cm, MW cut-off 10–12 kDa) and 5 mL of NaCl (0.9%) and 5 mL of NaHCO_3_ (0.5 M) were added. The mixture was placed inside the flask before the addition of 1.75 mL of pancreatin solution (800 U mL^−1^), bovine bile extract and porcine bile extract (1:1 *w*/*w* up to 10 mM total bile salts), and 14 µL of 0.3 M CaCl_2_. The mixture was kept under agitation at 37 °C for 2 h at 170 rpm. The collected digesta were placed on ice, moved to a freezer at −80 °C, and freeze-dried. Later, 10 mL of each sample was extracted from each digestion phase for additional analysis. The digested and undigested smoothies were kept at −80 °C until individual carotenoid and antioxidant levels and the inhibition of carbohydrate hydrolysing enzymes (α-amylase and α-glucosidase) analysis were determined. Equation (1) was used to determine the bioaccessibility of bioactive substances.

Bioaccessibility % = (BGC/BND) × 100(1)

The BGC (mg kg^−1^) was the content of the bioactive compound in the intestinal digesta, and BND (mg kg^−1^) was the bioactive compound content in the undigested sample.

### 2.10. Antioxidant Properties

Using the methods described by Seke et al. [2], the antioxidant capacity of VOP 1, VOP 3, and VOP 4 were assessed before and after digestion. At 517 nm, the absorbance for the DPPH test was determined. The result was expressed as the IC_50_ (mg mL^−1^). The radical scavenging ability of ABTS in smoothies was determined by measuring absorbance at 734 nm and expressing the results as IC_50_ (mg mL^−1^). A procedure outlined by Seke et al. [2] was followed to calculate the ferric-reducing antioxidant potential (FRAP) of the digested and undigested cowpea leaf smoothies. The Trolox equivalent antioxidant activity (TEAC)/100 g of cowpea leaf smoothies was calculated from the absorbance measured at 593 nm.

### 2.11. Inhibition of Carbohydrate Hydrolysing Enzymes (α-Amylase and α-Glucosidase)

The α-glucosidase inhibitory activity was determined using a microplate reader (CLARIOstar Plus BMG Labtec, Lasec, Cape Town, South Africa), as described by Moloto et al. [1]. The enzyme inhibitory activity was calculated and expressed as a percentage of α-glucosidase inhibition [26]. As described by Moloto et al. [1], the α-amylase inhibition of the extracts from the cowpea leaf smoothies was assessed using a microplate reader (CLARIOstar Plus BMG Labtec, Lasec, Cape Town, South Africa) monitored at 580 nm. The percentage of α-amylase inhibition was used to calculate enzyme inhibitory activity. Due to its efficiency, safety profile, favourable cardiovascular and metabolic effects, and capacity to be coupled with other antidiabetic drugs, metformin was used as a positive control in the study, being the first glucose-lowering drug of choice for treating persons with type 2 *diabetes mellitus*.

### 2.12. Glucose Uptake Assay

A method described by Chauke et al. [27] was followed to measure cellular glucose uptake. Muscle cells were placed in 96-well plates, cultivated for 4 days and incubated for a further 1, 4, and 6 h at 37 °C. The glucose test kit (KAT Medicals, Johannesburg, South Africa) was used to determine the amount of glucose in the medium. Insulin (100 M) was used as the positive control, and untreated cells as the negative control. The absorbance was then recorded at 540 nm, using a Multiskan GO (Thermo Scientific, Waltham, MA, USA). The results were presented as a percentage of the total amount of glucose consumed (mmol/L).

### 2.13. Statistical Analysis

The experiments were performed in triplicate and repeated twice, and the data were analyzed using one-way ANOVA in the statistical program Statistica data analysis software system (10) (Statsoft, Inc., Tulsa, OK, USA). The Fisher LSD test was performed to discover significant differences at *p*-values of 0.05. The linear correlations between phenolics and bioactivities were established using regression correlation coefficients. The nonlinear regression “dose-response inhibition” was used to determine the IC_50_ for DPPH and ABTS activities.

## 3. Results and Discussion

### 3.1. Physicochemical Properties of Cowpea Smoothies Fermented Using Ltp. plantarum 75

Food acidification is the key process involved in the lactic acid fermentation of food for preservation and safety by limiting the growth of spoilage and harmful bacteria in fermented foods. After 48 h of fermentation, the pH value of the cowpea smoothies decreased from 6.28 (VOP 1), 6.57 (VOP 3), and 6.51 (VOP 4), to 5.15 (VOP 1), 5.12 (VOP 3), and 5.05 (VOP 4) (Table 1). The pH decline observed in this study supports the findings by Managa. [15], who reported a pH drop in fermented chayote and pineapple smoothie after two days of fermentation. The reduction in the pH value of the smoothies was a key indicator of the fermentation progress, which relates to the production of organic acids [28]. Lowering the pH during LAB fermentation could hinder the growth of spoilage and pathogenic microorganisms, thereby aiding food preservation.

The total titratable acidity (TTA) increased gradually from 0.69 mg/mL (VOP 3), 0.75 mg/mL (VOP 4) and 0.78 mg/mL (VOP 1) to 1.98 mg/mL (VOP 1), 2.07 mg/mL (VOP 3) and 2.22 mg/mL (VOP 4) (Table 1). The gradual increase in total titratable acidity could be due to the metabolization of carbohydrates into organic acids, such as lactic acid being the predominant metabolic product in lactic acid bacteria fermentation [18]. Lower pHs during the fermentation of *Ltp. plantarum*-fermented emmer-based beverages supplemented with fruit juices have been reported, indicating *Ltp. plantarum* 75 is a vigorous heterofermenter that can thrive at low pH [29].

Total soluble solids (TSS) are significant quality markers concerning sweetness, often known as the sugar index (Magwaza and Opara [30]). The TSS content of smoothies fermented for 48 h significantly decreased in all of the cowpea smoothies (Table 1). The initial TSS content of the fermented cowpea smoothies was 1.70 (VOP 1), 1.51 (VOP 3), and 1.53 (VOP 4) °Brix but decreased to 0.82 (VOP 1), 0.63 (VOP 3), and 0.61 (VOP 4) °Brix in the fermented smoothies after two days (Table 1). The decrease in TSS levels during fermentation indicated the use of sugars in smoothies for metabolism, cellular development, and bioconversion into organic acid. This observation corroborates the claim of decreased TSS in *Ltp. Plantarum*-fermented beet juice after 72 h [31]. The same trend was also observed in mango juice [32]. However, an increase in TSS of the smoothies, as observed at 2 h of fermentation for all accessions, before an actual decrease might be attributed to polysaccharide breakdown into monosaccharide and oligosaccharide [33]. Furthermore, the total sugar content significantly decreased with increasing fermentation period across all cowpea accessions (Table 1). The decrease in the sugar content could, therefore, suggest that the fermented cowpea leaf smoothies could be a nutritional option for managing diabetic conditions.

### 3.2. Effect of Fermentation on the Ascorbic Acid Content of Three Different Cowpea Cultivar Leaf Smoothies

Table 2 shows the ascorbic acid (AA) concentration of fermented and unfermented cowpea leaf smoothies. An extended length of fermentation with *Ltp. plantarum* 75 resulted in a substantial rise in AA concentration. The unfermented samples had the lowest AA, whereas the two-day fermented smoothies had the highest AA. The AA content of the unfermented smoothies varied from 4.30 to 6.02 mg/100 g, whereas it ranged from 15.67 to 17.67 mg/100 g after two days of fermentation (Table 2). The results from this current research confirm a study that found an increase in AA when a vegetable–fruit beverage was fermented with *Ltp. plantarum* [34]. Several studies have investigated the effect of lactic acid fermentation on the vitamin C content of fruit and vegetable juices. Up to this point, their findings have indicated that the fermentation process has a variable impact on the ascorbic acid level of fermented foods. Predefined phases of fermentation as well as some lactic acid bacteria strains had a positive effect on AA content. After 12 h of fermentation of cashew apple juice [35] with *Ltp. plantarum* and *Lb. casei*, vitamin C levels increased and then remained constant after 48 h of fermentation. This observation could be attributed to microorganisms synthesizing ascorbic acid. Citrus juice fermented for 12 h had a vitamin C level that is comparable to conventional pasteurized juice [36]. Similarly, Znamirowska et al. [37] proposed that lactic acid bacteria could increase the vitamin C content of fermented dairy. 

In contrast, the utilization of *Lb. acidophilus* and *Lb. casei* as inoculum for fermentation culminated in a small decrease in vitamin C levels after 48 h of fermentation [37]. Following a few hours of fermentation, the ascorbic acid concentration of fermented juices from sweet lemon [38], prickly pears [39], and papaya [40] increased. The decrease in AA levels after some period of fermentation could be attributed to the increased activity of the ascorbate oxidase enzyme produced during lactic acid fermentation. It is not unexpected that bacteria that do not degrade ascorbic acid frequently have a protective effect, since ascorbic acid oxidizes in an aqueous solution, resulting in the creation of dehydroascorbic acid and hydrogen peroxide. Therefore, the observed increase in ascorbic acid may be due to decreased oxygen in the medium, and increased acidity since ascorbic acid is stabilized in acidic environments [41,42].

### 3.3. Colour Changes in Cowpea Leaf Smoothies after Fermentation

Table 3 shows the impact of fermentation on the colour of cowpea leaf smoothies. The luminosity (L*) value of the fermented and unfermented cowpea smoothies varied from 17.80 to 27.61, and a significant (*p* < 0.05) rise in the fermented smoothies (Table 3) was observed. The redness to greenness (a*) values of all of the accessions (VOP 1, VOP 3, and VOP 4) decreased significantly during fermentation, with a* values for the fermented and unfermented smoothies ranging from −6.91 to −3.90. The fermenting LAB metabolic activities could have caused enzymatic oxidation throughout the fermentation process resulting in the greenness-to-redness colour features in the fermented smoothies. The blue-to-yellow (b*) values increased significantly in the unfermented to the fermented smoothies after two days (Table 3). The ∆E relates to the colour difference of the fermented cowpea smoothies. After 48 h, the ∆E varied from 1.32 to 3.89. VOP 1 (1.32) had the lowest ∆E and was substantially different from VOP 3 (2.67) and VOP 4 (3.89) fermented smoothies. The fermentation duration had a significant effect on the colour parameter values for all smoothies. The lesser colour change in the VOP 1-fermented smoothie at 48 h may be due to the slower fermentation taking place in the cultivar compared to others, as shown by a lower TTA (Table 1). This might be due to the presence of polysaccharides in the cultivar that needed to be broken down into simple sugars before utilisation by *Ltp. plantarum* 75, thus reducing enzymatic degradation in fermented smoothies. *Ltp. plantarum* has been reported as a powerful facultative heterofermenter of food substrates [43]. However, the increased ∆E levels (VOP 3 and VOP 4) in fermented smoothies might be attributed to the auto-oxidation of the polyphenolic components [44].

### 3.4. Microbial Counts in Fermented and Unfermented Cowpea Leaf Smoothies

**Figure 1:**
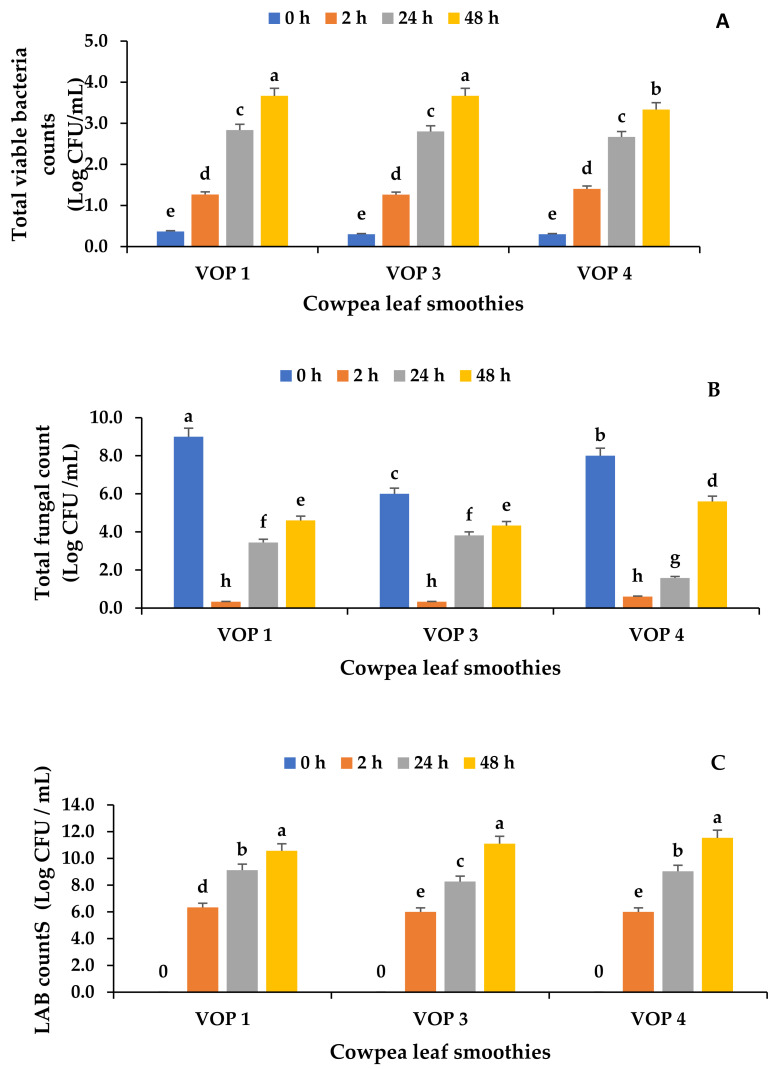
The microbial load in fermented and unfermented cowpea leaf smoothies. (**A**) Total viable bacterial count, (**B**) total fungal count, and (**C**) lactic acid bacteria count. The data presented on the graphs consist of average quantities ± SD of three independent samples. Different letters on the bars represent statistically significant differences (*p* < 0.05). VOP 1, VOP 3, and VOP 4 are different cowpea leaf cultivar smoothies, and units are shown as colony-forming units per millilitre (CFU/mL).

To evaluate the variations in microbial quality in the fermented smoothies, the yeast and mould count, total viable bacteria count, and lactic acid bacteria count were evaluated. As shown in Figure 1A, the total viable bacteria counts were lowest in the unfermented but pasteurised smoothies from all cultivars and were not significantly different (*p* ≥ 0.05). The total bacteria counts of fermented smoothies from different cultivars were not significantly different to each other at 2, 24, and 48 h of fermentation, except VOP 4 at 48 h of fermentation (*p* ≤ 0.05). The yeast count was highest in the unfermented smoothies and lowest in the 2 h-fermented smoothies (Figure 1B). However, the yeast and bacterial counts were within the acceptable limits (Log 6 CFU/mL) for beverages [18]. No pathogens, such as *E. coli*, *Salmonella* spp. and *Staphylococcus aureus*, were detected in the smoothies (Table S2). Presumptive lactic acid bacteria count ranged from 7 Log CFU/g after 2 h to 10 Log CFU/g after 48 h (Figure 1C). As expected, there was an increase in the presumptive LAB count in the fermented cowpea leaf smoothie after 48 h in all cultivars (VOP 1, VOP 3, and VOP 4). The increase in the LAB count might be due to the evolution of *Ltp. plantarum 75* through the utilisation of organic sugars in smoothies causing fermentation. *Ltp. plantarum* prefers glucose and lactose as carbon sources and can adapt to a variety of environments, which explains its versatility in fermentation [45].

### 3.5. Sensory Evaluation of Unfermented and Fermented Cowpea Leaf Smoothies

**Figure 2:**
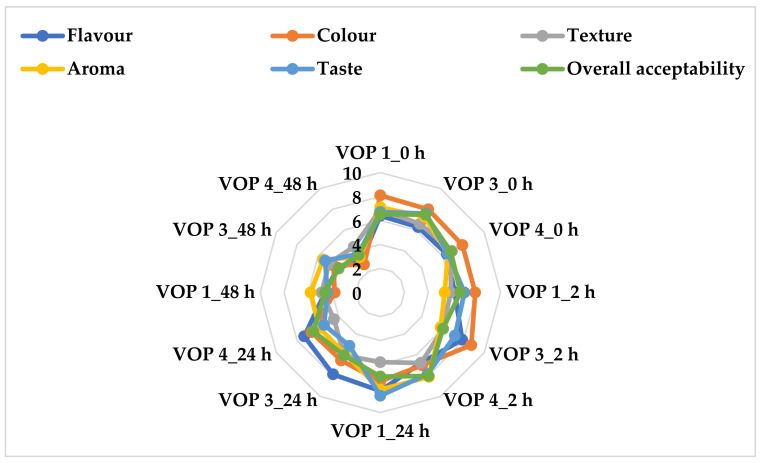
Sensory evaluation of fermented and unfermented cowpea leaf smoothies. VOP 1, VOP 3, and VOP 4 = cowpea cultivar leaf smoothies; LAB 75 = *Ltp. Plantarum* 75; VOP 1_0 h, VOP 3_0 h, and VOP 4_0 h = pasteurized and unfermented smoothies; VOP 1_2 h, VOP 3_2 h, and VOP 4_2 h = 2 h-fermented smoothies; VOP 1_24 h, VOP 3_24 h, and VOP 4_24 h = 24 h-fermented smoothies; VOP 1_48 h, VOP 3_48 h, and VOP 4_48 h = 48 h-fermented smoothies.

Figure 2 shows how the organoleptic properties of fermented and unfermented cowpea leaf smoothies were obtained from different cultivars. The colour perception of the unfermented and fermented smoothies varied from browning leafy vegetable colour, (2.70) in VOP 4 at 48 h, to dark-green leafy vegetable colour, (8.75) in VOP 3 after 2 h. There were no significant differences between the unfermented and 24 h-fermented smoothies; however, the overall acceptability of smoothies fermented for 48 h was considerably lower than the 2 and 24 h counterparts. VOP 3 and VOP 4 were the least acceptable. The declining green colour of leaf smoothies may be due to the oxidation of the samples [46] during acidity and fermentation. Lactic acid fermentation makes use of the oxidation and reduction processes in the transformation of molecules found in substrates. Typically, during fermentation, pyruvates are reduced to lactate by oxidizing NADH to NAD+ [10]. When compared to the other samples, the VOP 1 fermented smoothie after 24 h (8.20) had a highly acceptable flavour, while the VOP 4 fermented smoothie after 48 h had the least (3.10), as shown in Figure 2. The unfermented VOP 1 smoothie had a considerably better taste in terms of acceptability than the other unfermented samples, whereas the 48 h-fermented VOP 4 had the lowest acceptable taste (Figure 2). As fermentation progressed, the perception of flavour diminished. This could be due to the utilization of the sugar substrates that resulted in the fast depletion of sugars in the smoothies, thereby leaving the *Ltp. plantarum* 75 to utilise phenolics as a substrate for its bioconversion. Lactic acid bacteria have been reported to hold the potential as probiotics by utilising metabolites such as phenolics for their metabolic activities [10]. The aroma of VOP 1 fermented for 24 h was found to be substantially more acceptable than the other fermented smoothies. Overall, the 24 h-fermented VOP 1 smoothies can be recommended regarding organoleptic acceptability.

### 3.6. Total Phenolic Content (TPC) and Antioxidant Activities of Unfermented and Fermented Cowpea Leaf Smoothies

The total phenolic content of the unfermented and fermented smoothies from different cowpea leaf cultivars is shown in Table 4. The TPC was noted to be significantly higher in the unfermented (249.80 mg/100 g DW) and 2 h-fermented VOP 1 smoothie (249.90 mg/100 g DW), and was lowest in the 48 h-fermented VOP 3 smoothie (172.23 mg/100 g DW). The VOP 3 and VOP 4 fermented smoothies had a highly significant (*p* < 0.05) reduction in TPC at 48 h at 19.7% and 15.25%, respectively (Table 4), whilst there was an 11.11% reduction in the TPC of the VOP 1 smoothie after 48 h of fermentation (Table 4). The period of fermentation had a significant effect on the TPC of the cowpea smoothies. The reduction in the TPC of the fermented smoothies is consistent with the findings of Hashemi et al. [10], based on sweet lemon juice fermented by *Ltp. plantarum*. Similarly, Yang and co-workers noted a decrease in the TPC of a vegetable–fruit beverage with an increase in the fermentation period [34]. The decrease could be attributed to phenolic component degradation due to the low sugar content of the smoothies (Table 1). Some lactic acid bacteria may degrade phenols to continue growing; this action may result in a decrease in overall phenolic concentration [34,47]. 

Table 4 presents the FRAP antioxidant activity of cowpea leaf smoothies before and after fermentation. Fermentation by *Ltp. plantarum* 75 increased the antioxidant activity of the cowpea leaf smoothies to 300.41, 81.61, and 83.69 mmol TEAC/100 g DW, in VOP 1, VOP 3, and VOP 4, respectively, compared to the unfermented cowpea leaf smoothies. VOP 3 showed the lowest antioxidant activity while VOP 1 had the highest FRAP, DPPH, and ABTS antioxidant activity after fermentation. The FRAP, DPPH, and ABTS activities positively correlate with the TPC, with coefficient values of R^2^ = 0.98, R^2^ = 0.49, and R^2^ = 0.74, respectively (Table S3). 

### 3.7. Effect of Fermentation on the Carotenoid’s Profiles in Cowpea Leaf Smoothies

The main free xanthophyll in the fermented cowpea smoothies was lutein, and it was significantly higher in concentration than the carotenoids during the fermentation period (Table 5). The content of lutein decreased with increasing fermentation period in all of the cowpea cultivars; VOP 1 (33.48 to 42.70%), VOP 3 (38.48 to 46.77%) and VOP 4 (41.03 to 46.49%) after fermentation for 48 h (Table 5). A similar trend was noted with zeaxanthin, α-carotene, 9-cis-β-carotene, and all-trans β-carotene. The decrease in carotenoid content after fermentation was in the order VOP 4 > VOP 3 > VOP 1 (Table 5). In general, carboxylic acids created during fermentation are reported to provide an acidic environment in which carotenoids are considered to be unstable due to their potential hydrolyzation into free xanthophylls and fatty acids, which could be further converted or degraded [27]. The unfermented smoothies indicated the highest carotenoid content in VOP 1 (148.71 mg/100 g), VOP 3 (123.29 mg/100 g), and VOP 4 (107.09 mg/100 g). The lowest degradation rate of total carotenoid content was noted in VOP 1 fermented for 24 h. However, the highest percentage loss was noted in VOP 3 with 41.96% at 24 h of fermentation, which increased to 50.26% after 48 h of fermentation. A degradation of total carotenoids during fermentation in sweet potato [46], vegetable juice [48], and yellow lantern pepper soup [27] fermented with *Ltp. plantarum* have been reported. The degradation of total carotenoids suggests fermentation with *Ltp. plantarum* 75 could impact carotenoid stability. Therefore, the reduction in carotenoids during fermentation could be attributed to bacterial metabolism that results from the change in substrate temperature, pH, and produced metabolites [48]. However, the degree of metabolism by fermenting microorganisms is determined by the strain utilized, the substrate, and the fermentation conditions [48]. 

### 3.8. Effect of In Vitro Digestion on Total Phenolic Content and Antioxidant Activity of VOP 1 Fermented Cowpea Smoothie

Table 6 indicates the effect of gastrointestinal digestion on total phenolic compounds of the VOP 1 cowpea leaf smoothie fermented for 24 h. The VOP 1 cultivar was selected based on its high antioxidant activity, retained carotenoid profile and total phenolic content after 24 h of fermentation. The TPC of the undigested smoothie (223.97 mg/100 g DW) was significantly higher than that of the digested smoothie. The TPC of the gastric digesta in the fermented smoothies was reduced significantly when compared to the equivalent undigested smoothie, with a bioaccessibility of 86.07%. (Table 6). The TPC levels in the intestinal phase (335.25 mg/100 g DW) were greater than the undigested smoothie (223.97 mg/100 g DW) and had the highest bioaccessibility (149.57%). The bioaccessibility at the dialysis phase was 30.68%. TPC decreased significantly throughout the dialysis phase when compared to the undigested, gastric, and intestinal digesta. A similar pattern was observed in digested apple cultivars, such as Jonaprinz, Jonagold, and Golden, with total phenol levels in the intestinal phase being higher but less conspicuous than in the gastric phase [49]. Similarly, during the in vitro digestion of fermented chayote leaf and pineapple smoothies, the intestinal phase had higher levels of total polyphenol content than the gastric digesta [15]. The observed increase in the TPC of the intestinal digesta compared to the gastric digesta may be due to an increased release of phenolics bound to the matrix due to the activity of the intestinal digestive enzyme (pancreatin), or due to the phenolic interaction with cell wall carbohydrates such as pectin present in the smoothies, thereby obstructing the phenolic compound solubilization during gastric digestion.

The FRAP activity was significantly high (*p* < 0.05) in the intestinal digesta (345.46 μmol TEAC/100 g) compared to the undigested samples (320.78 μmol TEAC/100 g) and the gastric digesta (304.89 μmol TEAC/100 g) (Table 6). Furthermore, the ABTS and DPPH radical scavenging activities were found to be significantly high in the undigested and the intestinal digesta (Table 6). Tagliazucchi et al. [50] made a similar report on phenolic-rich grape extracts that offered better protection and high ABTS antioxidant capacity at the intestinal phase than in the gastric phase. The observed tendency could be attributed to the higher phenolic contents of the undigested sample and intestinal digesta [50]. According to reports, pH fluctuations between the gastric and intestinal phases also altered antioxidant activity by increasing the antioxidant capacity of phenolics [51]. Phenols can function as reducing agents, hydrogen donors, and singlet oxygen-reducing agents due to their redox properties, all of which increase their capacity as natural antioxidants. Additionally, the quantity and placement of hydrogen-donating hydroxyl groups on the aromatic ring of phenol molecules affect their ability to scavenge free radicals and act as antioxidants [52].

### 3.9. Effect of In Vitro Digestion on the C_2_C_12_ Glucose Uptake of Fermented VOP 1 Cowpea Leaf Smoothies

**Figure 3:**
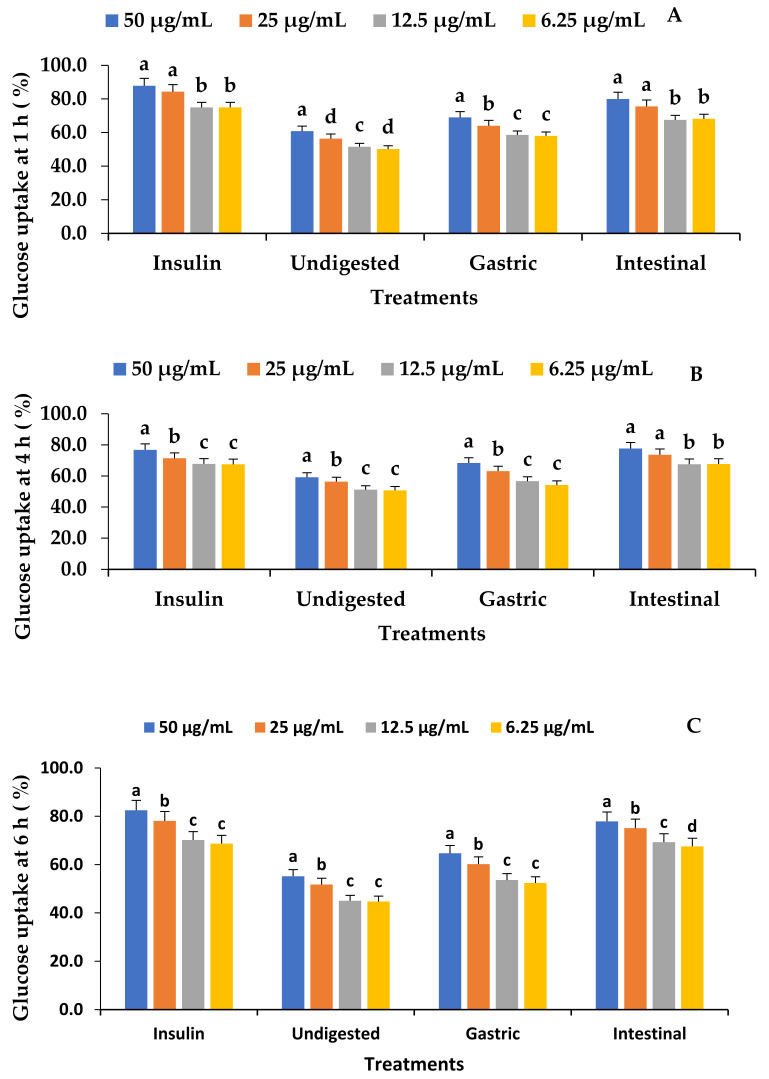
The effect of the in vitro digestion of VOP 1 cowpea leaf smoothies fermented by *Ltp. plantarum* 75 on muscle cell C_2_C_12_ glucose uptake at 1, 4, and 6 h. The data presented on the graphs consist of average quantities ± SD of three independent samples. The different letters on the bars represent statistically significant differences per treatment (*p* < 0.05). (**A**) 1 h C_2_C_12_ glucose uptake (%), (**B**) 4 h C_2_C_12_ glucose uptake (%), and (**C**) 6 h C_2_C_12_ glucose uptake (%).

The glucose uptake of the VOP 1 fermented smoothies at 1 h, 4 h, and 6 h indicated an improved glucose uptake activity (Figure 3). The intestinal digesta indicate a significantly high glucose uptake relative to the undigested samples and the gastric digesta, with a trend of 50 µg/mL > 25 µg/mL > 12.5 µg/mL, and 6.25 µg/mL. However, the glucose uptake was not significantly different between 6.25 and 12.5 µg/mL (*p* ≥ 0.05) except in the undigested and intestinal fractions at 1 h and 6 h of glucose uptake, respectively (*p* ≤ 0.05). The insulin treatment of the skeletal muscle cells resulted in an increase that was equal to the intestinal digesta (Figure 3). The enhanced translocation and movement of *GLUT4* to the plasma membrane are primarily responsible for the stimulation of glucose absorption [53]. Moloto et al. [1] observed that leaf extracts of cowpea cultivar VOP 1 dramatically elevated the *GLUT4* gene to a level comparable to insulin therapy. This could be an indicator of increased glucose absorption by C_2_C_12_ cells stimulated by the pool of phenolic chemicals in the VOP 1 fermented smoothies. The antioxidant and anti-inflammatory effects of polyphenols have been linked to the ability to reduce the risk of developing diabetes by inhibiting tyrosine phosphatase. This activates tyrosine phosphorylation and suppresses hepatic glucose output through processes that interact with the cell membrane receptors. Green tea epigallocatechin gallate (EGCG) has been shown to regulate the activity of the cell surface receptor, tyrosine kinases (*RTK*), such as insulin receptors (*InsR*) and insulin-like growth factor receptors (*IGFR*) [54,55]. In addition, some polyphenols such as flavonoids are known for their capacity to chelate metals, which inhibits the generation of free radicals that are catalysed by metals [56].

### 3.10. Effect of In Vitro Digestion on the α-Glucosidase and α-Amylase Inhibitory Capacity of Fermented VOP 1 Cowpea Leaf Smoothies

**Figure 4:**
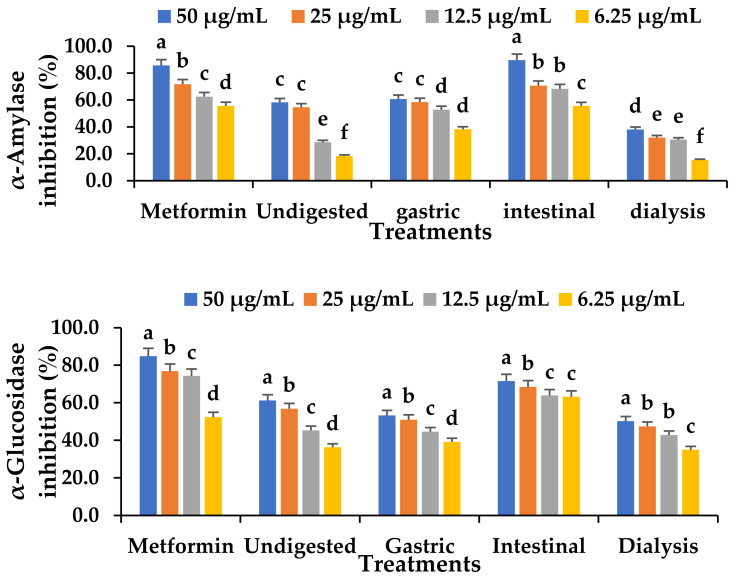
The effect of the in vitro digestion of *Ltp. plantarum* 75 fermented VOP 1 cowpea leaf smoothie on α-glucosidase and α-amylase inhibition using muscle cells C_2_C_12_ for 24 h. The data presented on the graphs consist of average quantities ± SD of three independent samples. Different letters on the bars represent statistically significant differences per treatment (*p* < 0.05).

The major enzymes for digesting carbohydrates, α-amylase and α-glucosidase, have been identified as therapeutic targets for the management of postprandial hyperglycaemia, which manifest in type 2 *diabetes mellitus*. The α-glucosidase and α-amylase inhibitory activity of the digested and undigested VOP 1 fermented smoothie are presented in Figure 4. The inhibitory capacity of α-glucosidase was greater in the intestine phase than in the undigested, gastric, and dialysis phases (Figure 4). The diabetic-lowering abilities of polyphenols have been linked to their anti-inflammatory and antioxidative properties, in addition to their excellent insulin signalling abilities [57]. 

A comparable study has linked the high polyphenol content after digestion to a strong α-glucosidase inhibition capacity observed in the intestinal phase compared to the gastric phase [58]. Pomegranate extracts have been noted to inhibit α-glucosidase activities in the intestinal phase [59]. Similarly, Rusak et al. [59] reported the abilities of matcha tea and sencha green teas in inhibiting α-glucosidase in the intestinal phase. The TPC positively correlates with the α-glucosidase inhibition capacity (R^2^ = 0.95) in the intestinal phase (Table S3), indicating that an increase in phenolics consequently increased the α-glucosidase inhibition. Similarly, the α-amylase was significantly inhibited by the intestinal digesta (*p* < 0.05) compared to the undigested, gastric, and dialysed digesta (Figure 4). With a trend of 50 µg/mL > 25 µg/mL > 12.5 µg/mL > 6.25 µg/mL at different concentrations of α-glucosidase and α-amylase, glucose absorption was promoted in the intestinal compartment at high concentrations. Consequently, blocking the enzymes may help to lessen the rate of glucose absorption and ameliorate postprandial hyperglycaemia [60,61]. A study reported a significant inhibitory effect of α-glucosidase and α-amylase in fermented vegetable juice [62]. Koh et al. [63] demonstrated a significantly higher α-glucosidase inhibition activity in an *Lb. mali*-fermented pumpkin-based beverage. 

### 3.11. Evolution of Lactic Acid Bacteria in Fermented VOP 1 Cowpea Leaf Smoothie after Simulated Gastrointestinal Digestion

**Figure 5:**
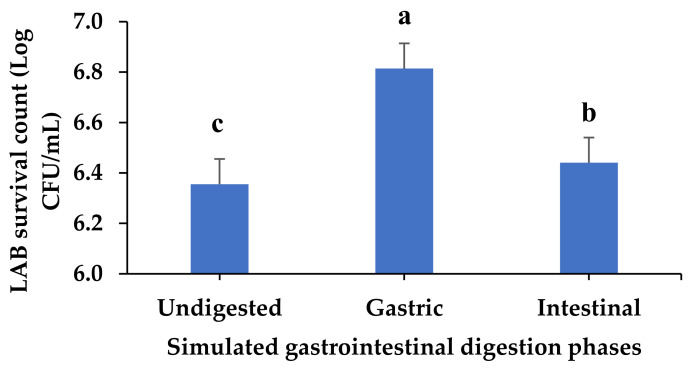
Surviving LAB count in simulated gastrointestinal digesta from fermented VOP 1 cowpea leaf smoothie. The data presented on the graphs consist of average quantities ± SD of three independent samples. The different letters on the bars represent statistically significant differences (*p* < 0.05); Log CFU/mL = logarithmic colony-forming unit per millilitre of smoothies.

The recommended characteristics of a probiotic microbe include gut survival, persistence in the host, and proof of safe human consumption [62]. The surviving LAB population across digestion phases was investigated. LAB counts ranged from 6.3 Log CFU/mL in the undigested sample to 6.8 Log CFU/mL in the gastric phase, which had a significantly high population of LABs (*p* < 0.05) (Figure 5). Many consumed microorganisms die due to the harsh acidity conditions in the stomach; however, acid-resistant strains, such as *Lactobacillus* spp., *Bifidobacterium* spp., and *Streptococcus* spp., can survive [64]. A similar result regarding the significantly high surviving LAB count in the *Lb. acidophilus*-fermented beverage subjected to gastric digestion [65] supports the observation in this study. 

Furthermore, lactic acid bacteria in orange juice with a pH of 3.80 were found to be more resistant to the gastrointestinal environment [65]. According to Chen et al. [66], the *Ltp. plantarum* PM153 strain showed the best adhesion ability and high survival in gastric fluids. Similarly, it was found that fermented beverages made from chickpeas and coconut had a considerably greater LAB survival rate in the gastric phase [67]. The acid tolerance ability among the *Lactobacillus* genus could be due to the presence of a continuous gradient between their extracellular and intracellular pH [68]. Moreover, the presence of glucose in an acid environment has been shown to improve *Lactobacillus* probiotic life by providing the needed ATP pool, thus allowing optimal H^+^ extrusion via F0F1-ATPase, and, therefore, boosting the capacity to survive in simulated gastric juice [69]. The cause of the decline in bacterial survival in the simulated intestinal digesta could be attributed to bile salts, which are components of bovine bile, and the pancreatin solution, which most impairs the viability of microorganisms. Bile salts can modify cellular homeostasis and macromolecular stability by affecting phospholipids and membrane proteins. A study by Mesquita et al. [67] demonstrated the reduction in *Ltp. plantarum* viability after exposure to pancreatic juice.

## 4. Conclusions

This study demonstrated that the fermentation of cowpea smoothies from VOP 1, VOP 3, and VOP 4 cultivars with *Ltp. Plantarum 75* improves the AA and antioxidant capacity (FRAP, DPPH, and ABTS) due to an increase in the total phenolic compounds and carotenoid constituents after fermentation. VOP 1 smoothies fermented for 24 h had acceptable sensory properties. *Ltp. Plantarum* 75 was viable and could survive the harsh conditions of the gastrointestinal tract; hence, it could be used as a probiotic in cowpea leaf smoothies. VOP 1 cowpea leaf smoothies have bioaccessible polyphenols and antidiabetic effects at gastric and intestinal phases, as shown by its increased glucose uptake by the C_2_C_12_ cells. Moreover, VOP 1 digesta had higher α-glucosidase and α-amylase inhibition activity and could be effective in managing diabetes. Thus, the lactic acid bacteria fermentation of cowpea leaf smoothies could improve the bioaccessibility of carotenoids, phenolic compounds, and antioxidants, and improves its potential as a vehicle for nutraceuticals to manage pathological conditions in the body. Future research should focus on the bioaccessibility of phenolic compounds in CaCo_2_ cells and the prebiotic potential of polyphenols in cowpea leaves should be investigated.

## Figures and Tables

**Table 1:** Changes in physicochemical properties of *Ltp. plantarum* 75-fermented cowpea (*Vigna unguiculata*) leaf smoothies from three different accessions.

Cowpea Cultivars	Treatment	h	pH	TTA (mg/mL)	TSS (° BRIX)	TS (30 mg/100 g)
VOP 1	Unfermented	0	6.28 ± 0.21 ^a^	0.78 ± 0.05 ^c^	1.30 ± 0.28 ^c^	1.58 ± 0.16 ^b^
VOP 3	Unfermented	0	6.57 ± 0.44 ^a^	0.69 ± 0.02 ^c^	1.35 ± 0.15 ^c^	1.35 ± 0.11 ^b^
VOP 4	Unfermented	0	6.51 ± 0.12 ^a^	0.75 ± 0.01 ^c^	1.43 ± 0.47 ^c^	1.96 ± 0.23 ^a^
VOP 1	LAB 75	2	6.18 ± 0.33 ^a^	1.05 ± 0.04 ^c^	1.70 ± 0.08 ^b^	1.01 ± 0.27 ^b^
VOP 3	LAB 75	2	6.4 ± 0.41 ^a^	1.11 ± 0.08 ^c^	1.51 ± 0.11 ^c^	0.96 ± 0.13 ^c^
VOP 4	LAB 75	2	6.4 ± 0.31 ^a^	1.12 ± 0.05 ^c^	1.53 ± 0.17 ^a^	1.66 ± 0.27 ^b^
VOP 1	LAB 75	24	5.66 ± 0.82 ^b^	1.29 ± 0.08 ^b^	1.01 ± 0.11 ^d^	0.62 ± 0.02 ^c^
VOP 3	LAB 75	24	5.33 ± 0.10 ^b^	1.50 ± 0.10 ^b^	1.16 ± 0.05 ^d^	0.37 ± 0.01 ^d^
VOP 4	LAB 75	24	5.21 ± 0.61 ^b^	1.80 ± 0.10 ^b^	1.00 ± 0.08 ^d^	0.12 ± 0.01 ^e^
VOP 1	LAB 75	48	5.15 ± 0.03 ^c^	1.98 ± 0.10 ^a^	0.82 ± 0.11 ^e^	0.33 ± 0.04 ^d^
VOP 3	LAB 75	48	5.12 ± 0.01 ^c^	2.07 ± 0.19 ^a^	0.63 ± 0.15 ^e^	0.22 ± 0.01 ^d^
VOP 4	LAB 75	48	5.05 ± 0.02 ^c^	2.22 ± 0.29 ^a^	0.61 ± 0.05 ^e^	0.09 ± 0.01 ^e^
LSD *			0.63 **	0.80 **	0.37 ***	0.30 **

Values are mean ± standard error of means; means followed by a different letter within the column are significantly different * *p* ≤ 0.05, ** *p* ≤ 0.01, and *** *p* ≤ 0.001. TTA = titratable acidity, TSS = total soluble solids, TS = total sugars, LAB 75 = *Ltp*. *Plantarum* 75, VOP 1, VOP 3, and VOP 4 = cowpea cultivar leaf smoothies, and LSD = least significant difference.

**Table 2:** Ascorbic acid contents of *Ltp. plantarum* 75-fermented and unfermented cowpea leaf smoothies from different cultivars.

Cowpea Cultivar	h	Treatment	AA (mg/100 g)
VOP 1	0	Unfermented	6.02 ± 0.01 ^e^
VOP 3	0	Unfermented	4.30 ± 0.48 ^g^
VOP 4	0	Unfermented	5.20 ± 0.35 ^f^
VOP 1	2	LAB 75	6.33 ± 0.50 ^e^
VOP 3	2	LAB 75	4.32 ± 0.01 ^g^
VOP 4	2	LAB 75	5.52 ± 0.57 ^f^
VOP 1	24	LAB 75	16.10 ± 0.01 ^b^
VOP 3	24	LAB 75	12.38 ± 0.01 ^d^
VOP 4	24	LAB 75	15.33 ± 5.48 ^c^
VOP 1	48	LAB 75	17.67 ± 0.48 ^a^
VOP 3	48	LAB 75	15.67 ± 1.96 ^c^
VOP 4	48	LAB 75	16.34 ± 0.48 ^b^
LSD *			1.65 ***

Values are mean ± standard error of means; means followed by a different letter within the column are significantly different * = *p* ≤ 0.05, *** *p* ≤ 0.001. AA = ascorbic acid, h = hour, LAB 75 = *Lactiplantibacillus plantarum* 75, LSD = least significant difference, and VOP 1, VOP 3, and VOP 4 = cowpea cultivars leaf smoothies.

**Table 3:** Colour changes in fermented and unfermented cowpea leaf smoothies from three different cowpea cultivars.

Accession	h	Treatment	L*	a*	b*	∆E
VOP 1	0	Unfermented	17.80 ± 0.24 ^i^	−5.81 ± 0.13 ^b^	11.66 ± 0.02 ^c^	
VOP 3	0	Unfermented	22.16 ± 0.92 ^e^	−6.25 ± 0.29 ^a^	12.15 ± 0.10 ^b^	
VOP 4	0	Unfermented	23.97 ± 0.19 ^e^	−6.91 ± 0.32 ^c^	10.88 ± 0.21 ^d^	
VOP 1	2	LAB 75	20.33 ± 0.37 ^b^	−5.55 ± 0.06 ^e^	10.03 ± 0.21 ^c^	1.03 ± 0.62 ^d^
VOP 3	2	LAB 75	23.86 ± 0.23 ^f^	−6.09 ± 0.30 ^a^	12.03 ± 0.35 ^b^	1.05 ± 0.58 ^d^
VOP 4	2	LAB 75	25.14 ± 0.05 ^a^	−6.88 ± 0.29 ^d^	10.11 ± 0.23 ^d^	1.00 ± 0.10 ^d^
VOP 1	24	LAB 75	21.90 ± 0.08 ^g^	−4.07 ± 0.18 ^c^	12.69 ± 0.05 ^b^	3.83 ± 0.24 ^b^
VOP 3	24	LAB 75	25.73 ± 0.50 ^c^	−5.98 ± 0.30 ^c^	10.92 ± 0.26 ^d^	2.44 ± 0.85 ^c^
VOP 4	24	LAB 75	27.64 ± 0.22 ^b^	−5.70 ± 0.31 ^b^	11.78 ± 0.58 ^b^	5.75 ± 0.65 ^a^
VOP 1	48	LAB 75	21.70 ± 0.90 ^g^	−4.62 ± 0.23 ^a^	9.96 ± 0.36 ^e^	1.32 ± 0.08 ^d^
VOP 3	48	LAB 75	24.57 ± 0.44 ^d^	−3.68 ± 0.24 ^a^	12.55 ± 0.68 ^b^	2.67 ± 0.29 ^c^
VOP 4	48	LAB 75	27.61 ± 0.14 ^b^	−3.90 ± 0.08 ^a^	12.88 ± 0.09 ^a^	3.89 ± 0.48 ^b^
	LSD *		1.26 **	2.71 ***	1.31 ***	0.22 **

Values are mean ± standard error of means; means followed by a different letter within the column are significantly different at * *p* ≤ 0.05, ** *p* ≤ 0.01, *** *p* ≤ 0.001, and ns = not significant. LSD = least significant difference; L* = degree of lightness; a* = red-to-green component; b* = yellow-to-blue component; ∆E = total colour change, h = hour; LAB 75 = *Lactiplantibacillus plantarum* 75; VOP 1, VOP 3, and VOP 4 = cowpea cultivar leaf smoothies.

**Table 4:** Antioxidant properties and total phenol content of fermented and unfermented cowpea leaf smoothies obtained from three cultivars.

Cultivars	h	Treatment	Total Phenols	Loss	FRAP	DPPH	ABTS
			**(mg/100 g DW)**	**(%)**	**(mmol TEAC/100 g DW)**	**(IC50 μg/mL)**	**(IC50 μg/mL)**
VOP 1	0	Unfermented	249.80 ± 68.26 ^a^		171.79 ± 30.25 ^b^	1.14 ± 0.06 ^b^	30.41 ± 3.05 ^a^
VOP 3	0	Unfermented	214.49 ± 62.12 ^b^		167.32 ± 30.08 ^b^	1.50 ± 0.05 ^a^	24.52 ± 2.85 ^b^
VOP 4	0	Unfermented	205.17 ± 52.32 ^b^		163.38 ± 40.55 ^b^	1.38 ± 0.08 ^a^	15.47 ± 1.25 ^d^
VOP 1	2	LAB 75	249.90 ± 61.59 ^a^	0.04	170.54 ± 35.26 ^b^	1.15 ± 0.02 ^b^	20.20 ± 2.56 ^c^
VOP 3	2	LAB 75	211.78 ± 58.12 ^b^	1.26	160.54 ± 52.32 ^b^	1.48 ± 0.05 ^a^	2.96 ± 0.59 ^g^
VOP 4	2	LAB 75	205.45 ± 45.28 ^b^	0.13	160.25 ± 45.36 ^b^	1.39 ± 0.05 ^a^	10.95 ± 2.45 ^f^
VOP 1	24	LAB 75	223.97 ± 43.92 ^b^	10.34	315.59 ± 45.13 ^a^	1.03 ± 0.08 ^b^	13.78 ± 4.25 ^e^
VOP 3	24	LAB 75	201.94 ± 64.88 ^b^	5.85	165.59 ± 33.32 ^b^	1.20 ± 0.04 ^b^	10.33 ± 1.56 ^f^
VOP 4	24	LAB 75	183.29 ± 45.97 ^c^	10.66	124.00 ± 24.25 ^c^	0.87 ± 0.01 ^c^	9.89 ± 1.28 ^f^
VOP 1	48	LAB 75	222.03 ± 34.98 ^b^	11.11	300.41 ± 45.05 ^a^	0.07 ± 0.01 ^e^	0.53 ± 0.01 ^i^
VOP 3	48	LAB 75	172.23 ± 48.10 ^c^	19.70	81.61 ± 14.24 ^d^	0.44 ± 0.01 ^d^	1.69 ± 0.21 ^h^
VOP 4	48	LAB 75	173.87 ± 43.80 ^c^	15.25	83.69 ± 9.05 ^d^	0.39 ± 0.01 ^d^	2.80 ± 0.18 ^g^
	LSD *		25.89 ***		41.88 **	0.18 *	4.20 ***

Values are mean ± standard error of means; means followed by a different letter within the column are significantly different at * *p* ≤ 0.05, ** *p* ≤ 0.01, and *** *p* ≤ 0.001. LSD * = least significant difference; LAB 75 = *Lactiplantibacillus plantarum* 75; VOP 1, VOP 3, and VOP 4 = cowpea cultivar leaf smoothies.

**Table 5:** The carotenoid profile of *Ltp. plantarum* 75 fermented and unfermented cowpea leaf smoothies from different cultivars (mg/100 g).

Cultivars	Treatment	Hours	Lutein	Zeaxanthin	α-Carotene	9-cis-β-Carotene	All-Trans β-Carotene	Total (Carotenoids)
VOP 1	Unfermented	0	99.88 ± 12.36 ^a^	2.19 ± 0.15 ^a^	4.63 ± 1.05 ^a^	3.76 ± 0.78 ^a^	38.27 ± 4.25 ^a^	148.71 ± 40.01 ^a^
VOP 3	Unfermented	0	85.04 ± 9.28 ^b^	2.22 ± 0.25 ^a^	4.55 ± 1.85 ^a^	3.64 ± 0.65 ^a^	27.84 ± 3.95 ^b^	123.29 ± 23.58 ^b^
VOP 4	Unfermented	0	70.38 ± 7.68 ^c^	2.29 ± 0.29 ^a^	5.07 ± 1.90 ^a^	3.12 ± 0.72 ^ab^	26.23 ± 2.86 ^b^	107.09 ± 19.08 ^c^
VOP 1	LAB 75	24	66.46 ± 8.01 ^d^	2.17 ± 0.26 ^a^	3.03 ± 1.02 ^b^	2.96 ± 0.52 ^b^	21.75 ± 3.58 ^b^	96.39 ± 14.02 ^d^
VOP 3	LAB 75	24	52.32 ± 4.32 ^e^	2.12 ± 0.30 ^a^	2.21 ± 1.26 ^c^	2.48 ± 0.48 ^b^	12.43 ± 2.96 ^c^	71.56 ± 10.58 ^e^
VOP 4	LAB 75	24	41.50 ± 5.09 ^e^	2.04 ± 0.21 ^a^	3.13 ± 0.98 ^b^	2.23 ± 0.59 ^b^	10.48 ± 2.54 ^c^	59.38 ± 8.36 ^f^
VOP 1	LAB 75	48	57.23 ± 4.18 ^e^	1.64 ± 0.15 ^b^	2.19 ± 0.19 ^b^	2.66 ± 0.60 ^b^	19.47 ± 1.95 ^b^	82.91 ± 11.25 ^e^
VOP 3	LAB 75	48	45.27 ± 5.00 ^e^	1.84 ± 0.17 ^b^	1.53 ± 0.20 ^c^	2.24 ± 0.54 ^b^	10.44 ± 2.47 ^c^	61.32 ± 8.96 ^f^
VOP 4	LAB 75	48	37.66 ± 4.89 ^f^	1.90 ± 0.18 ^b^	2.45 ± 1.28 ^b^	1.80 ± 0.48 ^c^	9.75 ± 1.05 ^c^	54.2 ± 7.49 ^f^
LSD *			14.28 **	0.25 *	1.35 **	0.44 **	9.80 ***	10.20 **

Values are mean ± standard error of means; means followed by a different letter within the column are significantly different at * *p* ≤ 0.05, ** *p* ≤ 0.01, and *** *p* ≤ 0.001; LAB 75 = *Lactiplantibacillus plantarum* 75; LSD = least significant difference; VOP 1, VOP 3, and VOP 4 = cowpea cultivar leaf smoothies.

**Table 6:** Antioxidants and total phenol activity of in vitro digested and fermented cowpea leaf smoothies from VOP 1 cowpea cultivar.

	Total Phenols	Bioaccessibility	FRAP	DPPH	ABTS
	(mg/100 g DW)	%	(μmol TEAC/100 g)	(IC50 μg/mL)	(IC50 μg/mL)
Undigested	223.97 ± 43.92 ^b^		320.78 ± 39.14 ^b^	1.03 ± 0.08 ^c^	30.78 ± 4.25 ^c^
Gastric	192.78 ± 35.68 ^c^	86.07 ± 11.86 ^b^	304.89 ± 48.21 ^c^	5.49 ± 0.44 ^b^	32.28 ± 11.48 ^b^
Intestinal	335.25 ± 65.32 ^a^	149.57 ± 28.21 ^a^	345.46 ± 36.76 ^a^	0.94 ± 0.01 ^c^	31.09 ± 9.23 ^c^
Dialysis	68.70 ± 5.90 ^d^	30.68 ± 1.67 ^c^	156.13 ± 24.92 ^d^	16.97 ± 3.85 ^a^	46.69 ± 10.58 ^a^
LSD *	30.87 ***	28.58 ***	15.68 ***	4.95 **	1.25 *

Values are mean ± standard error of means; means followed by a different letter within the column are significantly different at * *p* ≤ 0.05, ** *p* ≤ 0.01, and *** *p* ≤ 0.001; LAB 75 = *Lactiplantibacillus plantarum* 75; LSD = least significant difference; VOP 1, VOP 3, and VOP 4 = cowpea cultivar leaf smoothies; FRAP = Ferric-reducing antioxidant power; DPPH = 2,2-diphenyl-1-picrylhydrazyl; ABTS = 2,2′-azino-bis(3-ethylbenzothiazoline-6-sulfonic acid); TEAC = Trolox equivalent antioxidant capacity; and IC50 = the concentration of a drug or inhibitor needed to inhibit a biological process or response by 50%.

## Data Availability

Data supporting this research will be made available upon request.

## Supplementary Materials

The following supporting information can be downloaded at: https://www.mdpi.com/article/10.3390/foods12081701/s1, Table S1: The limit of detection (LOD), the limit of quantification (LOQ), and regression equations for carotenoid identification and quantification using HPLC–DAD; Table S2: The microbial load of viable bacterial count in fermented and unfermented cowpea leaf smoothies; Table S3: The correlation coefficient of total phenolic content with antioxidants and α-glucosidase of fermented and unfermented cowpea leaf smoothies obtained from three cultivars.
