# Supplementary material for: Influence of Fermentation on Functional Properties and Bioactivities of Different Cowpea Leaf Smoothies during In Vitro Digestion
Source: Foods. 2023 Apr 19;12(8):1701. doi: 10.3390/foods12081701 (PMC10137366; doi:10.3390/foods12081701)
Supplement: Supplementary file 1 [file foods-12-01701-s001.zip › foods-2308213-supplementary materials/Supplementary Table 1 19 APRIL 2023.pdf]

Supplementary Table S1: Carotenoid identification and quantification using HPLC-UV-DAD

Carotenoids	Regression equation	R^2	LOQ (µg/L)	LOD (µg/L)
Lutein	Y=94894x + 834002	0,95	80.3	26,5
9-cis-β-carotene	Y=86352x - 255284	0,99	4,49	1,48
all-trans β-carotene	Y=30127x + 3E+06	0,98	54,6	18.6
zeaxanthin	Y=68769x - 740682	0,98	22,1	7,31
α-carotene	Y=26242x - 47686	0,99	13.3	4.4

* Limit of detection (LOD) and Limit of quantification (LOQ)
