# Supplementary material for: Influence of Fermentation on Functional Properties and Bioactivities of Different Cowpea Leaf Smoothies during In Vitro Digestion
Source: Foods. 2023 Apr 19;12(8):1701. doi: 10.3390/foods12081701 (PMC10137366; doi:10.3390/foods12081701)
Supplement: Supplementary file 1 [file foods-12-01701-s001.zip › foods-2308213-supplementary materials/Supplementary Table 2 19 APRIL 2023.pdf]

Supplementary Table 2: . The microbial load of viable bacterial count in fermented and unfermented cowpea leaf smoothies.

Accession	h	Treatment	XLD<br>Log CFU/mL	MCA
VOP 1	0	Unfermented	0.00±0.00[a]	0.00±0.00[a]
VOP 3	0	Unfermented	0.00±0.00[a]	0.00±0.00[a]
VOP 4	0	Unfermented	0.00±0.00[a]	0.00±0.00[a]
			0.00±0.00[a]	0.00±0.00[a]
VOP 1	2	LAB 75	0.00±0.00[a]	0.00±0.00[a]
VOP 3	2	LAB 75	0.00±0.00[a]	0.00±0.00[a]
VOP 4	2	LAB 75	0.00±0.00[a]	0.00±0.00[a]
			0.00±0.00[a]	0.00±0.00[a]
VOP 1	24	LAB 75	0.00±0.00[a]	0.00±0.00[a]
VOP 3	24	LAB 75	0.00±0.00[a]	0.00±0.00[a]
VOP 4	24	LAB 75	0.00±0.00[a]	0.00±0.00[a]
			0.00±0.00[a]	0.00±0.00[a]
VOP 1	48	LAB 75	0.00±0.00[a]	0.00±0.00[a]
VOP 3	48	LAB 75	0.00±0.00[a]	0.00±0.00[a]
VOP 4	48	LAB 75	0.00±0.00[a]	0.00±0.00[a]
	LSD*		0.00[ns]	0.00[ns]

Values are mean ± standard error of means; means followed by a different letter within the column are significantly different at * p ≤0.05, ** p ≤0.01, *** p ≤0.001, and ns = not significant. LSD = least significant difference; XLD = Xylose Lysine Deoxycholate agar , MCA =MacConkey agar VOP 1, VOP 3, and VOP 4 = cowpea cultivar leaf smoothies.
