# Supplementary material for: Influence of Fermentation on Functional Properties and Bioactivities of Different Cowpea Leaf Smoothies during In Vitro Digestion
Source: Foods. 2023 Apr 19;12(8):1701. doi: 10.3390/foods12081701 (PMC10137366; doi:10.3390/foods12081701)
Supplement: Supplementary file 1 [file foods-12-01701-s001.zip › foods-2308213-supplementary materials/Supplementary Table 3 19 APRIL 2023.pdf]

Supplementary Table 3: The correlation coefficient of total phenolic content with antioxidants and α-glucosidase of fermented and unfermented cowpea leaf smoothies obtained from three cultivars.

	FRAP	DPPH	ABTS	α-glucosidase
	(μmol TEAC/100 g)	(IC50 μg/mL)	(IC50 μg/mL)	%
TP **(mg/100 g DW)**	0.98***	0.49*	0.74**	0.95***

Significantly different at * $p \leq 0.05$, ** $p \leq 0.01$, *** $p \leq 0.001$, ; FRAP = Ferric-reducing antioxidant power; DPPH = 2,2-diphenyl-1-picrylhydrazyl; ABTS = 2,2′-azino-bis(3-ethylbenzothiazoline-6-sulfonic acid); TEAC = Trolox equivalent *antioxidant* capacity; and IC50 = the concentration of a drug or inhibitor needed to inhibit a biological process or response by 50%.
